# Differences in Uveal Melanoma Age-Standardized Incidence Rates in Two Eastern States of Australia Are Driven by Differences in Rurality and Ultraviolet Radiation

**DOI:** 10.3390/cancers13235894

**Published:** 2021-11-23

**Authors:** Melissa Chalada, Charmaine A. Ramlogan-Steel, Bijay P. Dhungel, Amanda Y. Goh, Samuel Gardiner, Christopher J. Layton, Jason C. Steel

**Affiliations:** 1 School of Health, Medical and Applied Sciences, Central Queensland University, Rockhampton, QLD 4701, Australia; m.chalada@cqu.edu.au (M.C.); c.ramlogan-steel@cqu.edu.au (C.A.R.-S.); 2 Gene and Stem Cell Therapy Program Centenary Institute, University of Sydney, Camperdown, NSW 2050, Australia; b.dhungel@centenary.org.au; 3 Faculty of Medicine and Health, The University of Sydney, Sydney, NSW 2006, Australia; 4 Faculty of Medicine, Greenslopes Clinical School, The University of Queensland, Greenslopes, QLD 4120, Australia; amanda.goh@uq.net.au; 5 Clinical Research Centre, Sydney Local Health District, Camperdown, NSW 2050, Australia; Samuel.Gardiner@health.nsw.gov.au; 6 LVF Ophthalmology Research Centre, Translational Research Institute, Brisbane, QLD 4102, Australia

**Keywords:** uveal melanoma, cutaneous melanoma, ultraviolet radiation, Australia, Queensland, Victoria

## Abstract

**Simple Summary:**

Uveal melanoma (UM) is a rare form of melanoma originating in the eye. Unlike cutaneous melanoma (CM), the role of ultraviolet radiation (UVR) in UM aetiology is still unresolved. UM has a high incidence in Australia. Epidemiological analyses revealed heterogeneity in UM incidence between two eastern Australian states, Queensland (QLD) and Victoria (VIC). It was found that QLD has a 21% higher incidence of UM than VIC and, in fact, has one of the highest incidences in the world. A weak south-to-north trend in incidence along the eastern Australian coast is seen, and rural areas have a 24% greater burden than major city areas in both the states. The two states are similar demographically, but differ socially, industrially and latitudinally. This is important because it could indicate a minor UVR role in UM incidence, especially in QLD. Preventative measures by sun-protective behaviours may be important, especially in the northeastern Australian demographic.

**Abstract:**

Uveal melanoma (UM) is the second-most-common melanoma in humans and has a high age-standardized incidence rate (ASR) in Australia. Regional patterns of UM ASRs in Australia are unknown. The aim of this study was to determine and compare UM ASRs in two geographically disparate eastern states, Queensland (QLD) and Victoria (VIC), by using cancer registry data that was obtained from 2001 to 2013. World-standardized UM ASRs and incidence-rate ratios (IRRs) were calculated. Higher UM ASR was also observed in anterior UM compared to posterior UM ASR. UM ASR remained unchanged from 2001 to 2013 in QLD but decreased in VIC. A south-to-north latitude trend in UM ASR along the east of Australia is weakly evident, and rural populations have higher UM ASRs than major city populations in both states. Differences in ultraviolent radiation (UVR) susceptibility, indigenous populations, social behaviours, chemical exposure, and socioeconomic status could all be contributing to differences in UM rates between QLD and VIC and between rural compared to major city areas. It is possible that a minority of cases in QLD and VIC might be prevented by sun-protective behaviours. This is important, because these findings suggest that QLD, which is already known to have one of the highest cutaneous melanoma (CM) ASRs in the world, also has one of the highest UM ASRs.

## 1. Introduction

Uveal Melanoma (UM) is the most common intraocular melanoma in humans, arising from melanocytes of the iris, choroid or ciliary body in the eye. UM age-standardized incidence rate (ASR) appears to be elevated in Australia, with world-standardized ASR ranging from 6.2 to 8.0 per 1,000,000 person-years in men and 5.2 to 6.1 per 1,000,000 person-years in women [1,2]. This is high compared to other countries, such as Canada (men, 3.9; women, 3.5) [3], the United States (men, 5.1; women, 4.4) [4], and England (men, 4.7; women, 4.2) [2] per 1,000,000 person-years. In fact, analyses of 11 European populations, four American populations, and three Australasian populations showed that the Australian continent was reported to have one of the top five UM ASRs in the world [2]. However, the ASR of UM in Australia has not been assessed since the 1980s and 1990s [1,2], and there is little published data on the influence of regionality on Australian UM ASR. 

Queensland (QLD) and Victoria (VIC) are states with the most northern and southern latitudes of the Australian mainland, respectively, and are found at similar longitudes. QLD is 7.5 times the size of VIC [5] but has comparable population (3.9 million compared to 4.9 million in 2006, respectively) [6,7], with the majority of QLD classified as remote while VIC is mostly regional [8]. Both states have a single capital centre. Demographical proportions of different sexes and races in the two states are both similar to the Australian average, but QLD has a substantially greater proportion of Aboriginal and Torres Strait Islander people than VIC (3.3% compared to 0.6% in 2006) [6,7]. QLD also has a greater proximity to the equator (latitudes −29.0° to −9.8°) than VIC (−39.1° to −34.0°), contributing to a greater average daily solar exposure [9] and UV index [10]. Thus, the similar population sizes and demographics allow a relatively controlled ecological study of the impact of latitude and by inference UVR on UM epidemiology.

The comparison of two states with very similar populations, health systems, and governing variables provides a unique setting for the scientific investigation of population-wide factors associated with UM ASRs. Indeed, there already exist substantial differences in cancer burdens between the pair of states: QLD has one of the highest Cutaneous Melanoma (CM) ASRs in the world [11], and between 2008 and 2012, CM was the second-most-common cancer in QLD men (under prostate cancer) but only the third-most-common in VIC men (under prostate cancer and lung cancer) [12]. 

The aim of this study was therefore to use the unique characteristics of this state pair to compare UM ASR and incidence-rate ratios (IRR) patterns between 2001 to 2013 and to reflect on possible factors that drive differences in UM ASR. 

## 2. Materials and Methods

### 2.1. Obtaining UM Case Data for VIC and QLD

UM data from 2001 to 2013 was obtained from the QLD Cancer Registry (extracted 3 March 2016) and the Victorian Cancer Registry (extracted 8 March 2016). This encompassed all ICD-O-3 C69 sites (eye and adnexa), from 2001 to 2013, with ICD-O-3 morphology 872-879. Information on sex, year of birth, year of diagnosis, postcode inhabitancy at time of diagnosis, and site of origin were provided for both states. In this study, melanomas of the “conjunctiva” (C690) and “lacrimal gland” (C695), which have sometimes been classed as “ocular melanomas” in the past [1,13,14,15], are considered by the authors to be “mucosal melanomas” and are therefore excluded from analysis. Similarly, cases classed as “orbit, not otherwise stated” (C696), “overlapping lesion of eye and adnexa” (C698), and “eye, not otherwise stated” (C699) were excluded in this study to avoid the accidental inclusion of protruding mucosal or CMs. Cases classed as “corneal” melanomas (C691) were included in the analyses because they were likely to be ciliary body or iris melanomas that were viewable through the clear cornea [16]. Similarly, cases classed as “retinal” melanomas (C692) were considered to be likely misclassifications of choroidal melanomas [2,4,17] and were also included. C692 and C693 were pooled as “posterior UM” in this study (choroidal melanoma), while C691 and C694 were pooled as “anterior UM” (ciliary body or iris melanoma).

### 2.2. Geographical Grouping of UM Cases

In order to assess the relationship between UM ASR, latitude, and remoteness, the data on postcode inhabitancy at the time of diagnosis was used. Cases without postcode information were excluded from comparison statistics involving rurality and latitude. Rurality was based on the Australian Bureau of Statistics (ABS) Remoteness Area (RA 2011) level [8], i.e., major cities (mean Local Government Area RA 1 to 1.5), and rural areas (mean Local Government Area RA >1.5 to 5). Latitude bands were chosen based on the average annual noon clear-sky UV index band cut-offs and case numbers [10]. 

### 2.3. Statistics

ASRs per 1,000,000 person-years and their 95% confidence intervals (95% CI) were calculated for different geographical groups from 2001 to 2013 using annual population data from the ABS [18], and the world standard population. Time intervals and latitude bands were chosen to ensure the case numbers for that population were still sufficiently large (≥25 cases [19]) to determine accurate ASRs. Accuracy of rates was ensured by age standardization using 5-year age intervals [19]. Trends between UM ASR over time or latitude were calculated using bivariate correlations controlling for age (and sex where applicable). Correlation coefficients and their significance were only calculated when at least three independent variable points existed (e.g., QLD latitude bands 1, 2, 3). One-tailed t-tests were chosen over correlations when only two *x*-axis points were available as a result of insufficient case numbers (<25) for age standardization, per demographic, sex, time interval, and/or latitude band. Significance of correlations and t-tests were both determined using p values at the 0.05 significance level. The data was further analyzed by Poisson regression models to produce incidence rate ratios (IRRs) for characteristics such as rurality and latitude. Age and sex person-year exposure was included in every model to control for differences between the populations. IRRs were computed using per-sex, per-age-group, per-year population to account for increasing population over time. Statistics were performed using SPSS Statistics 26 or R 4.1.1.

## 3. Results

### 3.1. UM ASR and Case Demographics

From 2001 to 2013, there were 432 UM cases in QLD and 434 cases in VIC (see Table 1). Nine cases in QLD (1 male C692, 1 female C692, 5 female C693, and 2 female C694) did not have any postcode data and were excluded from ASR and IRR calculations involving latitude or rurality. The average age of UM diagnosis was significantly higher (*p* = 0.03) in VIC (62.8 years old) than in QLD (60.9 years old). 

**Figure 1:**
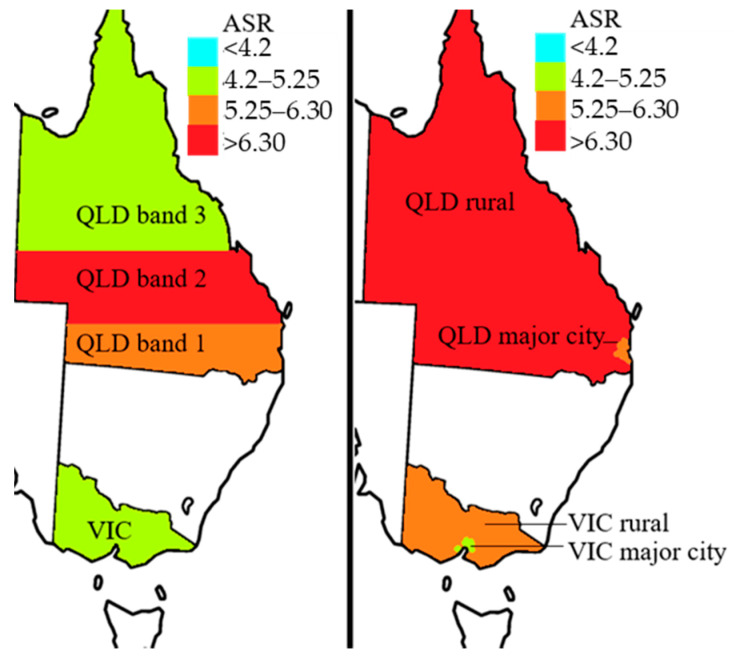
Summary of ASRs in different populations of QLD and VIC from 2001 to 2013. ASR, age-adjusted incidence rates per 1,000,000 person-years, standardized to world standard population. Major City, RA < 1.5; Rural, RA 1.5–5. VIC, latitude −39.1° to −34.0°; QLD band 1, latitude −29.0° to −26.7°; QLD band 2, latitude −26.7° to −21.6°; QLD band 3, latitude −21.6° to −9.8°.

Summaries of ASRs in Australia, VIC, and QLD are presented for UM in Table 2 and in Figure 1. QLD has a higher ASR of UM than VIC in both males (6.6 compared to 5.0 per 1,000,000, *p* < 0.001) and females (5.6 compared to 4.5 per 1,000,000, *p* = 0.01). When assessing individual sites of origins, QLD had a statistically higher ASR of anterior (ciliary body or iris) melanoma than VIC in both genders (1.2 compared to 0.7 per 1,000,000, *p* = 0.002). QLD also had a higher ASR of posterior (choroidal) melanoma in men (5.4) compared to VIC (4.2) per 1,000,000 (*p* = 0.05) but not in women (4.0 per 1,000,000 in QLD women compared to 3.7 per 1,000,000 in VIC women, *p* = 0.22).

Our ASR data is also supported by the IRR demonstrating a 21% (95% CI 6–39%, *p* = 0.005) higher incidence rate of UM in QLD than VIC when controlling for age, remoteness, and sex (Supplementary Table S1). Males have an 18% (95% CI 3–34%, *p* = 0.018) higher incidence than females when controlling for age and latitude and 17% (95% CI 3–34%, *p* = 0.020) higher incidence than females when controlling for age, state, and remoteness (Supplementary Table S1). 

### 3.2. UM Latitude Trends

The ASR of UM from VIC to QLD shows a positive correlation with latitude (*r* = +0.293, *p* = 0.03) only when the northernmost third of QLD is excluded (see Table 3). There is no latitude correlation in QLD (*r* = −0.113 change in ASR per 1,000,000 person-years per increase in latitude band, *p* = 0.42). When looking at the incidence rate we see that Central Queensland (QLD band 2) has a 74% greater incidence of UM (95% CI 40–115%, *p* < 0.001) than in VIC when controlling for age, sex, and year of diagnosis (see Figure 1 and Supplementary Table S2). Other latitude-band UM incidence rates from QLD are not statistically different to those from VIC.

### 3.3. UM Time Trends

**Figure 2:**
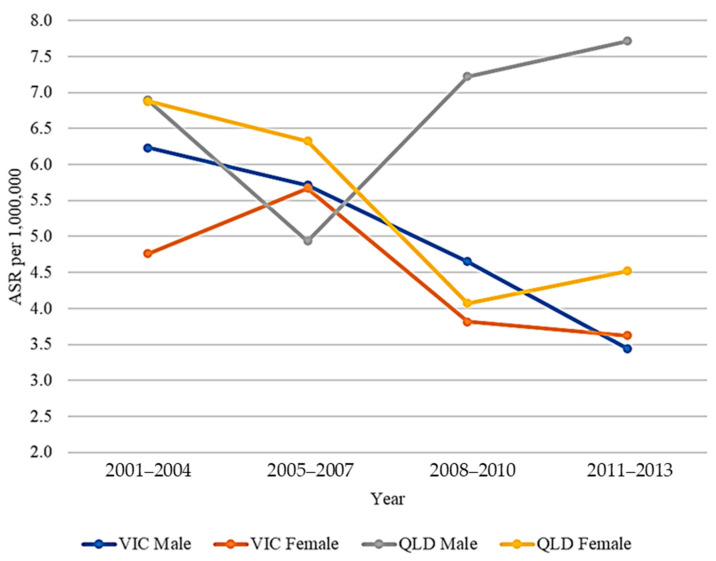
UM mean ASR over time in VIC and QLD from 2001 to 2013. Graph shows mean ASRs only. Bivariate regression of all ASRs against time, controlling for age: QLD male, *r* = +0.086, *p* = 0.48; QLD female, *r* = −0.215, *p* = 0.07; VIC male, *r* = −0.224, *p* = 0.06; VIC female, *r* = −0.149, *p* = 0.21.

UM time trends are presented in Figure 2. UM ASR did not change from 2001 to 2013 in QLD (*r* = −0.046, *p* = 0.59) but decreased in VIC (*r* = −0.189, *p* = 0.02). Comparisons of ASRs for younger and older VIC and QLD populations from 2001–2007 and 2008–2013 are presented in Table 4. UM ASR in older males (≥55 years old) was greater in 2008–2013 (5.2 per 1,000,000) than in 2001–2007 (3.6 per 1,000,000) in QLD (*p* = 0.03). In contrast, the ASR in older VIC males was lower in 2008–2013 (3.1 per 1,000,000) than in 2001–2007 (4.1 per 1,000,000). In younger cohorts (<55 years old), a lower UM ASR was seen in 2008–2013 than in 2001–2007 in QLD females (1.7 per 1,000,000 is less than 3.1 per 1,000,000 *p* = 0.03) but was not statistically different in QLD males (2.3 per 1,000,000 compared to 2.4 per 1,000,000, *p* = 0.46). In VIC, the UM ASR in younger individuals was not statistically different from 2001–2007 and 2008–2013 in either sex.

### 3.4. UM ASR in Major City and Rural Areas

All of the ASRs of UM in rural areas and major cities of VIC and QLD are summarised in Table 5. UM ASR is higher in rural areas than in major city areas of QLD (6.1 per 1,000,000 person-years compared to 5.8 per 1,000,000 person-years, *p* = 0.04). This is also seen in VIC (rural, 5.7 per 1,000,000 person-years compared to major city, 4.3 per 1,000,000 person-years, *p* = 0.04). When controlling for age and sex, the ASR of UM was still higher in QLD than in VIC in major city areas (5.8 per 1,000,000 person-years compared to 4.3 per 1,000,000 person years, *p* = 0.03) and rural areas (6.6 per 1,000,000 person-years compared to 5.7 per 1,000,000 person-years, *p* = 0.03). This is largely due to the higher rate of UM in QLD males in major cities and in QLD females in rural areas (see Table 5). This was backed up by our IRR data which showed that living in a rural rather than major city area increases the UM incidence rate by 24% (95% CI 8–43%, *p* = 0.002) when accounting for age, sex, and state (Supplementary Table S2).

## 4. Discussion

This is the first time that the ASRs of UM in QLD and VIC have been directly compared. The (world-standardized) ASR of UM from 2001 to 2013 was higher in QLD (males, 6.6; females, 5.6, per 1,000,000 person-years) than in VIC (males, 5.0; females, 4.4, per 1,000,000 person-years). The UM ASR in QLD (but not VIC) is also higher than the Australian average reported previously (6.2 per 1,000,000 person-years in men and 5.2 per 1,000,000 person-years in women [2]). The data indicate that QLD may have one of the highest UM ASRs in the world when compared to world-standardized rates reported previously, including Canada (men, 3.9; women, 3.5) [3], the United States (men, 5.1; women, 4.4) [4], England (men, 4.7; women, 4.2) [2], and France (men, 5.5; women, 4.4) [2]. The only population with a higher reported world-standardized ASR than QLD is Denmark (men, 7.8; women, 6.5) [20]; while Scotland has UM ASRs on par with QLD (men, 6.9; women, 5.3) [2]. In contrast, VIC has similar UM rates to those reported for the United States [4]. The higher ASR of UM in QLD than in VIC is also consistent with higher ASR of other cancers in this time period, such as eye cancer as a collective (i.e., IDC-O-3 C69: malignant neoplasm of eye and adnexa, including uveal melanomas; other malignant melanomas; retinoblastomas; carcinomas; sarcomas; and other or unspecified malignant neoplasms) [12,21,22], CM [12,21,22], and cancer overall [12]. Interestingly, the difference is particularly marked in the higher anterior UM ASR in QLD (1.3, 95% CI 1.0–1.6 per 1,000,000) than in VIC (95% CI 0.5-0.9 per 1,000,000) (*p* = 0.002). The age of diagnosis is also lower (*p* = 0.03) in QLD (60.9 years old) than in VIC (62.8 years old). These differences in site of origin and age of diagnosis could implicate distinct aetiological drivers of UM in QLD and VIC.

One substantial difference between the two states compared is that VIC has an average annual noon clear-sky UV index of 6, while QLD UV index extends from 8 to 12 [10]. QLD also has greater daily solar exposure than VIC [9], and a greater portion of the state is engaged in outdoor occupations [6,7]. However, UM, unlike CM, is not considered by the World Health Organization (WHO) to be related to solar risk [23,24]. Around 95% of total UVR reaching the Earth’s surface is UV-A (wavelength 315–400 nm) while only 5% is UV-B (wavelength 280–315 nm) [25,26,27]. Although UV-B is more hazardous than UV-A radiation [28], UV-A radiation can cause damage by increasing oxidative stress [26] and has greater transmission through anterior ocular structures than UV-B [29,30]. Furthermore, it has been recently reported that a proportion of UM cases have molecular evidence of ultraviolet radiation (UVR) damage [31,32,33]. The higher ASR of UM in QLD and rural communities is therefore consistent with a hypothesis of UVR-induced UM. This is strengthened by the fact that the differences in ASRs are greater for anterior UMs: The anterior uveal structures (ciliary body and iris) do not have the protection of the UVR-absorbing lens like the posterior (choroidal) uvea, thus may be more likely to be susceptible to UVR carcinogenic changes [33,34].

The weak positive correlation (*r* = +0.293, *p* = 0.03) observed from VIC (latitude −39.1° to −34.0°) to the more populated parts of QLD (−29.0° to −21.6°) also suggests a role for UVR in pathogenesis. However, UM ASR falls in the remote, most UVR exposed areas of northern QLD. This, surprisingly, could also be argued to be supportive of a role of UVR in UM ASR along eastern Australia. This seems counterintuitive, but while UM ASR has not shown a south to north increase across the entire continent previously [1], the pattern directly mirrors the pattern displayed by CM, a UVR-related disease, which increases in ASR in Australia closer to the equator but which also demonstrates the unusual fall in ASR in the northernmost QLD latitudes [35,36,37]. As with CM, it is suggested this could be due to the high indigenous population closer to the equator (≥−22°) in QLD [38], the reduced UM risk in darker-skinned ethnicities worldwide [39,40], including Australia [41] and in QLD [42], and low levels of diagnosis in the remote north.

### 4.1. Rural Areas Have a Higher ASR of UM than in Major Cities of QLD and VIC

QLD has a higher ASR of UM than VIC even when considering rural and major city areas. Analysis of the state pairs showed that the ASR of UM is higher in rural areas than in major cities for VIC and for QLD. Higher ASR of UM in rural areas compared to urban areas has been reported in Australia-wide data previously [1]. 

Differences in ASR between rural and urban areas could be due to differences in UVR, pollution, occupational exposure to carcinogens, or socioeconomic status. In dense urban areas, the increased pollution and built environment can cause the absorption and scattering of UV light, reducing UVR exposure [43]. Differences in pollution types between city (industrial and automotive pollution [44]) and regional to remote areas (agricultural pollution such as pesticides [45]) may play a role. Agricultural workers have an increased incidence of some cancers [45]. The majority of QLD is used for natural vegetation grazing, while southeastern QLD and the majority of VIC are used for irrigated pastures, followed by cropping and forestry [46]. In rural areas of VIC, residual arsenic contamination still persists in soils, surface, and groundwaters from historical gold-mining activity [47], and residents of these areas have been reported to have higher risks of cancer [47]. In an assessment of the western half of VIC, regional areas with less accessibility to healthcare also had higher cancer incidence [48] and disadvantaged populations generally may also be more likely to have higher-cancer-risk lifestyles such as smoking, alcohol consumption, and poor diet [48]. However, no data on the relationship between UM ASR and socioeconomic status is available for Australia.

### 4.2. Possible Influence of Behavioural Patterns

This study observes the ASR of UM in two eastern Australia states, VIC and QLD, from 2001 to 2013 as recorded in the Victorian Cancer Registry and QLD Cancer Registry. These registries are reliable for a number of reasons. Firstly, reporting of all cancer diagnoses is mandatory in all Australian states and territories [49], and secondly, the national Australasian Association of Cancer Registries Executive Committee promotes the uniformity of reporting between different states [49]. Finally, false or repeated registrations in the national cancer databases are minimised by excluding benign, in situ, recurrent, or interstate duplicates [49]. 

Despite thorough case reporting, the ASRs of UM reported in this study may still be underestimations of the true values for several reasons. Nine QLD UM cases were excluded from the analyses due to a lack of postcode data, potentially creating a sampling bias in the latitude and rurality IRR investigations. In addition, the current study did not employ methods to investigate possible non-notifications by healthcare facilities. Despite mandatory reporting, 109 unnotified cases were discovered in Australia in the period of 1990 to 1998 [1]. Cases often missed by Australian registries included smaller or less-advanced cases (especially choroidal melanoma) that had been treated with eye-conserving procedures [1,2], and the registration of outpatient cases is also probably less complete than inpatients [1]. Finally, missing or ambiguous topography data for UMs can impact inclusion criteria. For example, Vajdic et al. (2003) reported that two ocular oncologists disagreed on the classification of 5% of Australian UM cases from 1990–1998 [1]. In the present study, the exclusion of melanoma cases classed as “orbit, not otherwise stated” (C696), “overlapping lesion of eye and adnexa” (C698), and “eye, not otherwise stated” (C699) from analysis may have inadvertently excluded some cases of uveal origin but does increase the likelihood that true UM were analysed. Differences in inclusion criteria likely contributes to the disparity between Australian UM ASRs reported by different studies [1,2].

This study relied on postcode at diagnosis for many of its analyses, and information on changes of inhabitancy of UM cases was not available. It can be observed through census data that high turnover or growth was greater in regional and remote areas over the study period than in major city areas, especially in QLD [50,51,52], while regional VIC displayed an overall greater stability in population than regional QLD [50,51]. The local government areas in QLD and VIC with the greatest population stability during the study period appeared to be clustered to major city areas [50,51]. Rather than being a major confounder though, on balance it is likely that population movements between higher and lower risk areas would have balanced themselves out to some degree across the sample. The analyses did not have data about other risk factors like race, genetic background, or immunosuppression. 

Unfortunately, data on the molecular profiles and pathological features of the diagnosed UM is not reported in the QLD or VIC cancer registries. This may not be surprising given that, in contrast to most other cancers, UM is frequently diagnosed without a confirmatory biopsy, and, instead, diagnosis and subsequent treatment is solely based on clinical findings [53]. This may change in the near future as recent studies have shown that both molecular and histopathological profiling of UM can predict disease-related mortality and as such may become a useful tool for clinical decision-making [54]. 

Our study benefits from the comparison of two latitudinally polar states with relatively similar demographics, latitude, and healthcare systems. Thus, an association be-tween UVR on UM ASR may be commented on. However, caution must be taken in this ecological study to not overstate the evidence for the relationship between UVR radiation and UM ASR, as this and similar ecological studies can only establish weak evidence towards this finding.

Finally, UM’s orphan status means that, statistically, a small change in the number of cases can have a large impact on the rate. Consequently, fluctuations in data should be interpreted with caution and more complicated analyses may not have their assumptions met in many samples of UM. 

## 5. Conclusions

QLD and VIC face different carcinogenic, demographic, and solar UVR burdens but represent grossly similar populations and living styles suitable for demographic comparison. Together, the identified factors may contribute to the higher ASR of UM in QLD, especially in men, in rural areas, and in central QLD. This is significant for 2 main reasons: 

Firstly, south-to-north latitude trends, higher rural ASR, and the substantially greater ASR of anterior UM in QLD than in VIC may all suggest a relationship between UM ASR and UVR in a proportion of cases. This agrees with anatomical, molecular, and epidemiological evidence that at least a small portion of UM cases are UVR-related [34]. 

Secondly, rural areas typically have less access to healthcare than major cities. This is particularly a problem in UM, which is usually asymptomatic in the early stages and often diagnosed incidentally by an optometrist [55]. This is in contrast to CM, which can be observed through whole-body skin examinations or may even be noticed macroscopically by a partner [56,57]. Currently, no early detection regimens exist for UM in QLD or VIC, and it may be necessary to analyze the need for these in rural settings with the higher UM rates reported in this analysis.

Our data has shown that from 2001 to 2013, QLD had a greater ASR of UM than VIC resulting in a 21% greater incidence. The UM ASR in QLD is also higher than average Australian rates and represents one of the highest UM rates in the world. Differences in UVR exposure, occupation, chemical exposure, and rurality could all plausibly contribute to the higher rates of UM in QLD than VIC, in men than in women, and in rural areas than in major city areas of both QLD and VIC. This means that awareness and early detection regimes should be considered, especially for these demographics.

## Figures and Tables

**Table 1:** Demographics of cases included in this study.

State	Sex	Site ^1^	No. of Cases	Mean Age of Diagnosis in Years (95% CI)
QLD	Female	C692 Retina	4	61.4 (59.0–63.7)
		C693 Choroid	146	
		C694 Ciliary Body/Iris	48	57.8 (52.1–63.4)
		C691 Cornea	2	
		Total	200	60.6 (58.5–62.8)
	Male	C692 Retina	5	60.7 (58.7–62.8)
		C693 Choroid	187	
		C694 Ciliary Body/Iris	35	62.3 (58.8–65.7)
		C691 Cornea	5	
		Total	232	61.1 (59.3–62.8)
VIC	Female	C692 Retina	3	62.3 (58.8–65.7)
		C693 Choroid	177	
		C694 Ciliary Body/Iris	27	56.5 (49.8–63.2)
		C691 Cornea	4	
		Total	211	62.3 (60.3–64.4)
	Male	C692 Retina	2	63.7 (61.5–65.8)
		C693 Choroid	189	
		C694 Ciliary Body/Iris	30	60.7 (58.7–62.8)
		C691 Cornea	2	
		Total	223	63.2 (61.2–65.1)

^1^ C692 and C693 were pooled as “posterior UM” in this study (choroidal melanoma), while C691 and C694 were pooled as “anterior UM” (ciliary body or iris melanoma).

**Table 2:** ASR ^1^ of UM in QLD and VIC from 2001 to 2013.

State	Melanoma ^2^	Male (95% CI)	Female (95% CI)	Persons (95% CI)
QLD	UM	6.8 (5.9–7.7)	5.5 (4.7–6.3)	6.1 (5.5–6.7)
VIC		5.0 (4.4–5.7)	4.4 (3.8–5.0)	4.6 (4.2–5.1)
QLD	Posterior UM	5.4 (4.6–6.2)	4.0 (3.3–4.6)	4.7 (4.2–5.2)
	Anterior UM	1.2 (0.8–1.6)	1.4 (1.0–1.8)	1.3 (1.0–1.6)
VIC	Posterior UM	4.2 (3.6–4.8)	3.7 (3.1–4.2)	3.9 (3.5–4.3)
	Anterior UM	0.7 (0.5–1.0)	0.7 (0.4–1.0)	0.7 (0.5–0.9)

^1^ ASRs are per 1,000,000 person-years, age-adjusted to world standard population. ^2^ C692 and C693 were pooled as “posterior UM” in this study (choroidal melanoma), while C691 and C694 were pooled as “anterior UM” (ciliary body or iris melanoma).

**Table 3:** UM ASR ^1^ along eastern Australia from 2001 to 2013.

Location	Latitude (°)	Male (95% CI)	Female (95% CI)	Persons (95% CI)	Average Annual Noon Clear-Sky UV Index 1979–2007 [10]
QLD Band 3	−21.6 to −9.8	─ ^2^	─ ^2^	4.3 (3.0–5.6)	10–12
QLD Band 2	−26.7 to −21.6	9.2 (7.0–11.4)	7.7 (5.6–9.9)	8.4 (6.9–9.9)	9
QLD Band 1	−29.0 to −26.7	6.7 (5.6–7.8)	4.8 (3.9–5.8)	5.8 (5.0–6.5)	8
VIC	−39.1 to −34.0	5.0 (4.4–5.7)	4.5 (3.8–5.1)	4.7 (4.3–5.2)	6

^1^ ASRs are per 1,000,000 person-years, age-adjusted to the world standard population. ^2^ Insufficient case numbers from 2001–2013 to accurately calculate age-standardized trends for individual sex.

**Table 4:** UM ASR ^1^ in QLD and VIC in different ages in 2001–2007 and 2008–2013.

State	Age (Years)	Period	Male (95% CI)	Female (95% CI)	Persons (95% CI)
QLD	<55	2001–2007	2.4 (1.6–3.2)	3.1 (2.2–4.0)	2.7 (2.2–3.3)
		2008–2013	2.3 (1.5–3.1)	1.7 (1.0–2.3)	2.0 (1.5–2.5)
	≥55	2001–2007	3.6 (2.7–4.5)	3.6 (2.7–4.4)	3.6 (3.0–4.2)
		2008–2013	5.2 (4.1–6.2)	2.7 (1.9–3.4)	3.9 (3.3–4.5)
VIC	<55	2001–2007	1.9 (1.3–2.5)	2.1 (1.4–2.7)	2.0 (1.6–2.5)
		2008–2013	1.6 (1.3–2.0)	1.6 (1.0–2.2)	1.6 (1.3–1.9)
	≥55	2001–2007	4.1 (3.2–4.9)	3.1 (2.4–3.8)	3.5 (3.0–4.0)
		2008–2013	3.1 (2.6–3.5)	2.1 (1.6–2.7)	2.8 (2.4–3.1)

^1^ ASRs are per 1,000,000 person-years, age-adjusted to world standard population.

**Table 5:** UM ASR ^1^ in QLD and VIC rural and major city areas from 2001 to 2013.

State	Region ^2^	Male (95% CI)	Female (95% CI)	Persons (95% CI)
QLD	Major City	6.9 (5.7–8.1)	4.7 (3.7–5.6)	5.8 (5.0–6.5)
	Rural	6.7 (5.4–8.0)	6.6 (5.2–7.9)	6.6 (5.7–7.5)
VIC	Major City	4.5 (3.7–5.3)	4.2 (3.5–4.9)	4.3 (3.8–4.8)
	Rural	6.2 (4.9–7.6)	5.2 (3.9–6.5)	5.7 (4.8–6.6)

^1^ ASRs are per 1,000,000 person-years, age-adjusted to world standard population. ^2^ Major City, RA < 1.5; Rural, RA 1.5–5.

## Data Availability

Data used in this research article is obtainable from the Victorian Cancer Registry and the Queensland Cancer Registry. Population data is freely available through the Australian Bureau of Statistics, https://stat.data.abs.gov.au/ (accessed on 22 November 2021).

## Supplementary Materials

The following are available online at https://www.mdpi.com/article/10.3390/cancers13235894/s1, Table S1: UM IRR ^1^ controlling for latitude in QLD and VIC from 2001 to 2013, Table S2: UM IRR ^1^ controlling for remoteness in QLD and VIC from 2001 to 2013.
