# Supplementary material for: Differences in Uveal Melanoma Age-Standardized Incidence Rates in Two Eastern States of Australia Are Driven by Differences in Rurality and Ultraviolet Radiation
Source: Cancers (Basel). 2021 Nov 23;13(23):5894. doi: 10.3390/cancers13235894 (PMC8657208; doi:10.3390/cancers13235894)
Supplement: Supplementary file 1 [file cancers-13-05894-s001.zip › cancers-1447661-supplementary.pdf]

Supplementary Tables

Supplementary Table S1. UM IRR [1] controlling for latitude in QLD and VIC from 2001 to 2013

**Demographic**		**IRR**	**95% CI**	***p*-Value**
Age	<55 years old	1.00	-	
	>55 years old	5.40	4.67–6.26	<0.001
Sex	Female	1.00	-	
	Male	1.18	1.03–1.34	0.018
Latitude Band[3]	VIC	1.00	0	
	QLD band 1	1.16	1.00–1.35	0.056
	QLD band 2	1.74	1.40–2.15	<0.001
	QLD band 3	1.01	0.74–1.33	>0.9

[1] IRR, Incidence Rate Ratio, computed using per-sex, per-age-group, per-year population. [2] VIC, latitude −39.1° to −34.0°; QLD band 1, latitude −29.0° to −26.7°; QLD band 2, latitude −26.7° to −21.6°; QLD band 3, latitude −21.6° to −9.8°.

Supplementary Table S2. UM IRR [1] controlling for remoteness in QLD and VIC from 2001 to 2013.

**Demographic**		**IRR**	**95% CI**	***p*-Value**
Remoteness[2]	Major City	1.00	-	
	Rural	1.24	1.08–1.43	0.002
State	VIC	1.00	-	
	QLD	1.21	1.06-1.39	0.005
Sex	Female	1.00	-	
	Male	1.17	1.03–1.34	0.020
Age	<55 years old	1.00	-	
	>55 years old	5.40	4.67–6.26	<0.001

[1] IRR, Incidence Rate Ratio, computed using per-sex, per-age-group, per-year population. [2] Major City, RA < 1.5; Rural, RA 1.5–5.
